# Enzymatic Treatments for Biosolids: An Outlook and Recent Trends

**DOI:** 10.3390/ijerph20064804

**Published:** 2023-03-09

**Authors:** Omar J. Quintero-García, Heilyn Pérez-Soler, Myriam A. Amezcua-Allieri

**Affiliations:** 1 Nanotechnology Division, CINVESTAV-IPN, Avenida Instituto Politécnico Nacional 2508, San Pedro Zacatenco, Mexico City 07360, Mexico; 2 Biomass Conversion Division, Instituto Mexicano del Petróleo, Eje Central Lázaro Cárdenas 152, San Bartolo Atepehuacan, Mexico City 07730, Mexico

**Keywords:** wastewater, biosolids, pretreatment, liquid state conversion, lignocellulosic materials, fungal enzymes

## Abstract

Wastewaters are nutrient-rich organic materials containing significant concentrations of different nutrients, dissolved and particulate matter, microorganisms, solids, heavy metals, and organic pollutants, including aromatic xenobiotics. This variety makes wastewater treatment a technological challenge. As a result of wastewater treatment, biosolids are generated. Biosolids, commonly called sewage sludge, result from treating and processing wastewater residuals. Increased biosolids, or activated sludge, from wastewater treatment is a major environmental and social problem. Therefore, sustainable and energy-efficient wastewater treatment systems must address the water crisis and environmental deterioration. Although research on wastewater has received increasing attention worldwide, the significance of biosolids treatments and valorization is still poorly understood in terms of obtaining value-added products. Hence, in this review, we established some leading technologies (physical, chemical, and biological) for biosolids pretreatment. Later, the research focuses on natural treatment by fungal enzymes to end with lignocellulosic materials and xenobiotic compounds (polyaromatic hydrocarbons) as a carbon source to obtain biobased chemicals. Finally, this review discussed some recent trends and promising renewable resources within the biorefinery approach for bio-waste conversion to value-added by-products.

## 1. Introduction

Wastewater is any water whose quality has been adversely affected by anthropogenic influence. Wastewater includes used domestic, urban, industrial, or mining liquid waste disposed of or mixed with previous waters (rainwater or natural water). Its importance is such that it requires systems of channeling, treatment, and eviction. Its improper treatment generates serious pollution problems.

Biosolids, or sewage sludge, are produced as a result of water pretreatment. The United States Environmental Protection Agency estimates that over 8 million tons (dry weight) of biosolids are produced annually; therefore, improvements in biosolids treatment and valorization are needed. Biosolids have a high water content (≥95%), which impedes their transport and disposal. Therefore, dewatering these wastes is paramount to reduce their volume and facilitate their handling and disposal [1]. Sludge mainly comprises microbial cells and biomass; specifically, biomass contains approximately 40% carbohydrates, 30% proteins, and 30% lipids in particulate form [2].

Biosolids contain high concentrations of organic matter, heavy metals such as Ni, Pb, Cr, and Zn, and various toxic contaminants (endocrine disruptors, pesticides, pharmaceuticals, and personal care compounds). The disposal of biosolids is a significant challenge. Conventional methods of biosolids disposal are landfilling, incineration, and dumping at sea. This leads to the accumulation of pollutants in environmental matrices, decreased land availability, and increased regulatory control [3]. In addition, some value-added products can be obtained from biosolids treatments, considering the chemical composition of biosolids (biomass). In order to address waste biomass disposal through biological development, a focus on the transformation of waste resources into value-added products is needed.

This document provides information regarding biosolid treatments, particularly the one related to the biological treatment to degrade biomass (cellulose, hemicellulose, and lignin) and xenobiotic compounds with similar structures (polyaromatic hydrocarbons such as benzopyrene, anthracene, and fluorene). At the end, future trends are described for enzymatic treatment, favoring the biorefinery approach for biomass valorization.

## 2. Methodology

A systematic literature review was performed using the main citation databases, such as Scopus and Web of Science (WoS). In the literature review, the following keywords were used: biosolids, sewage sludge, wastewater, pretreatment, liquid state conversion, biomass, lignocellulosic materials, fungal enzymes, polyaromatic hydrocarbons, and a biorefinery approach.

Multidimensional aspects of biosolid treatment and sustainability and a connected bibliometric analysis and systematic literature review on the subject “biological technologies for biosolids” were carried out by analyzing articles reported in peer-reviewed journals and citation databases. The specific goals of this literature screening were to identify (a) the state-of-the-art of peer-reviewed publications dealing with treatment for biosolids, (b) biological treatments (fungal enzymes), (c) value-added products from a wide variety of conversion processes and feedstocks (biomass), and (d) the biorefinery approach to valorize biosolids.

To fulfill the literature screening, search queries based on selective keywords and multilevel searching were strategically formulated for a period between 2005 and 2023.

A list of published articles on the above-mentioned research subjects was compiled and analyzed to extract the main available information. Table 1 summarized the main findings related to biosolids pretreatments; Table 2 included the commercial enzymes produced by ascomycetes; and Table 3 summarized the non-commercial enzymes produced by ascomycetes.

## 3. Biosolid Treatment

There are different alternatives for biosolid treatment (Table 1). Various researchers have developed several pretreatment processes, such as thermal, physical, biological, and combined processes, to increase the performance of the hydrolysis phase and achieve optimal stabilization of biosolids.

Stabilization of biosolids is accomplished by aerobic digestion, anaerobic digestion, and composting processes. These methods produce stable biosolids and value-added by-products such as bioenergy in the form of methane, biogas, and compost. Anaerobic digestion is the most commonly used process. During anaerobic digestion, the hydrolysis of organic matter present in the chemical composition of the activated sludge is considered the yield-limiting phase [4]. The most promising treatment is using enzymes. Below are the main enzymes that are involved.

**Table 1:** Different types of pretreatments for biosolids.

Pretreatment		Characteristics	Advantages	Disadvantages	By-Products Obtained
Physical	Pyrolysis [5,6]	Biomass is subjected to elevated temperature exceeding 400 °C in an inert environment, involving partial or total elimination of oxygen. It is classified into slow, fast, and flash pyrolysis. 50–70% biosolids volume reduction.	Uncomplicated technology that does not require complex equipment. Environmentally friendly. Different types of waste can be treated.	High energy parameters.Slow pyrolysis is associated with challenges that require previous remediation stages. In fast pyrolysis, low yields are obtained.	Biochar Bio-oil Biogas
	Mechanical [7,8]	The pretreatment reduces particle size and has two main effects: (i) increased biogas production by fragmentation of the substrate if the substrate has a high fiber content and low degradability (ii) faster digestion. The most commonly used methods are shredding and chopping, and the particle size should be 1–2 mm.	With increased anaerobic biodegradability, smaller particles increase the surface area of microorganisms, resulting in greater food availability for bacteria.	High energy consumption.	Biogas Methane
	Microwaves [2,9]	The disintegration of biosolids by heating. Cell lysis and fragmentation of the polymeric network occur due to the heat caused by the movement of polar molecules.	Cost-effective process. Instant process control. Fast and selective hea-ting. Low environmental emissions and lower energy consumption if the desired temperature is reached.	If temperature and pressure increase considerably: (i) increase in solubilization of the (ii) increase in energy consumption and process operating costs.	Biogas (increase in production ~1.38–1.46 times)
	Phase separation [3,10]	Reduction of particulate matter and increase of organic matter.	Increased accessibility of nutrients to methanogenic bacteria. Reduction of biosolids viscosity. Increased sludge solubilization.	High energy consumption. pH variations.	Methane
	Ultrasonication [11,12,13]	Pressure wave that causes cavitation in the solution. Hydromechanical shear forces that modify the structure of the biosolid.	Reduction of particle size.Increased sludge biodegradability. Increased solubilization of organic compounds and hydrolysis phase. Increased biological activity and enzyme release.	High energy consumption at industrial scale.	Biogas
Chemical	Advanced Oxidation Processes [14,15] Photocatalysis [4,16]	Increase in bioenergy from organic wastes. The disintegration of biosolids and degradation of their flocs by hydroxyl radicals.	The disintegration of the structure protects microorganisms from chemical reactions. Solubilization of substrates facilitates their digestion and improves their biodegradability. Increased efficiency of anaerobic digestion. Degradation of organic matter to a soluble form. Rapid processes.	High capital and operating/maintenance costs.	Methane
		A chemical reaction induced by the absorption of photons by a solid material is called a photocatalyst. When exposed to UV and solar radiation, the photocatalyst reacts to generate hydroxyl radicals.	The photocatalysts can be reused and recovered. No additional energy is required as the process is solar-powered. Fast processes.Increased solubilization of biosolids.	The limited state of the art.	Biogas Hydrogen
	Alkaline [17,18]	Solubilization of the cell wall favors the release of intracellular substances.	Increased efficiency of soluble chemical oxygen demand. It improved sludge digestibility. Lower carbon footprint.Lower energy consumption. Process performance depends on the type of sludge used.	Process performance depends on the type of sludge used.	Biogas Methane
	Acid [19,20]	In situ acid production through exploiting iron, sulfur, and ammonia-oxidizing microorganisms. Use inorganic acids (H_2_SO_4_, HCl) to eliminate pathogens and increase biosolids stabilization.	Low-cost process.Reduction of pathogens and organic particles. It increased soluble chemical oxygen demand.	Bioleaching method in the experimental phase. Use of large amounts of acid. Less sludge dewatering. Obtaining harmful by-products (furfural, hydroxymethylfurfural.	Methane Biogas
	Ozonation [21,22]	The cell wall is ruptured, and soluble substances inside the cell are released and assimilated by anaerobic organisms.	Increased disintegration of biosolids. Efficient process due to its high oxidative power.	High energy consumption. The prolonged time is required.	Methane Biogas
Biological	Anaerobic digestion by temperature steps [23,24]	Combines two temperature ranges, thermophilic and mesophilic. A separation of the microbiological phases occurs depending on the operating temperature. The combination of low and high temperatures accelerates feedstock hydrolysis and acidogenesis. Increased acetogenesis and symbiotic methanogenesis in the mesophilic phase. – 45–55% reduction in biosolids volume.	Increases anaerobic degradation. Increases particulate decomposition. Minimal energy consumption. Sterilization of pathogens. Increased hydrolysis of organic wastes Increased efficiency in biogas production.	No direct and parallel comparison of mesophilic and thermophilic phases. Limited analysis of pH and temperature values that optimize hydrolytic conversion.	Methane Biogas
	Immobilization [25]	Microorganisms, mainly bacteria, are fixed on support to stimulate their optimal growth and development. – 20% biosolids volume reduction.	Increases bacteria stability and protects them from damage caused by the external environment. Maintains high concentration and activity of microorganisms. Increases pollutant degradation efficiency. Highly cost-effective process since the immobilized microorganisms can be reused without significantly affecting the activity. Low-cost investment.Environmentally friendly.	Segregation of extracellular polymeric substances (EPS). EPS hinders the dispersion of substrates in the matrix and minimizes the surface area for their subsequent disintegration, which reduces the potential of the pretreatments used.	Methane
	Biostimulation [13]	Biological stabilization of biosolids. Continuous nutrient addition. Biostimulation processes enhance biosolids bioremediation by producing hydrolytic enzymes from indigenous microorganisms.	Increased activity of the indigenous microbial consortium. Increased degradation of contaminants Increases biosolids stabilization. Promotes hydrolysis, a rate-limiting step in anaerobic digestion.	Selection of a stimulating agent readily available to microorganisms and does not inhibit pollutant degradation.	Biogas (production increased from 7 to 76%) Methane
	Microaerobic hydrolysis [26,27]	The facultative microorganisms consume the added oxygen. The reduction of the organic matter remains constant throughout the process.	Increased solubilization of chemical oxygen demand (COD). Degradation of poorly degradable compounds under microaerobic conditions. Increased concentration of soluble proteins and total sugars. Increased activity of hydrolytic and acidogenic bacteria. Reduction of hydrogen sulfide concentration. Simple and easy-to-operate technology.	The studies concerning this process are in the first phase. The effects of inoculum parameters, pretreatment time, and oxygen concentration are yet to be explored.	Methane (114% yield increase) Biogas
Combined process [9,28]		Combination of more than one physical, chemical, or biological process.	Increased solubility of biosolids. Reduction of limitations associated with individual pretreatment processes.	Studies on the application of combined processes are at an early stage.	Biogas Methane

This work discloses the relevant characteristics of physical, chemical, and biological methods for the pretreatment of biosolids. Some present a series of advantages and disadvantages of application and the obtaining of secondary products with added value for use and exploitation as energy resources (Table 1).

The cost for each treatment may vary, depending on the machinery, installation, maintenance, operation, quantity, and complexity of the material to be processed. In the case of pyrolysis, a capital investment of approximately 623 thousand dollars is required, which involves the plant, installation, supplies, and operation. The cost can be raised by the application of temperatures ranging from 400 to 800 °C to transform the waste into liquid, solid, or gaseous by-products with commercial exploitation value. Another implemented method is waste incinerators. These industries require an investment capital of approximately 100,000 dollars, which is unsustainable for many developing countries.

Alternatively, the use of biological systems such as enzymes promises a lower economic cost compared to other methods since the acquisition of organisms, characterization, and production of commercial and non-commercial enzymes range from around 300 to 500 dollars [29]. Due to the above, this does not mean that biological treatments present greater viability due to production costs, but rather that they can be an alternative in reducing investment and elaborating hybrid methods (physical-biological; chemical-biolo-gical), which can jointly carry out the treatment of solids, generating a lower economic and environmental impact.

### 3.1. Enzymatic Treatment

The *enzymes* are organic molecules that act as catalysts for chemical reactions, i.e., they accelerate the reaction rate. They are commonly found as proteins in nature and modify the reaction rate without affecting the balance of the reaction, since an enzyme causes a chemical reaction to take place at a higher speed, as long as it is energetically possible. In these reactions, enzymes act on molecules called substrates, which are converted into different molecules called products. Almost every process in cells needs enzymes to occur at significant rates. Enzyme-mediated reactions are called enzymatic reactions.

As part of the characterization of enzymes, the evaluation of their activity against a substrate plays an important role and is generated under factors such as temperature, type of substrate, and biological aspects that may be involved in the activation or inhibition of the catalyst. The presence of these macromolecules is a biotechnological advantage in the degradation of pollutants and is relevant to determining how efficient the system is for practical use. Lignocellulolytic enzymes and oxidoreductases produced by mesophilic and extreme fungi have demonstrated enzymatic activity in rigorous conditions such as acidic pH, hypersalinity, low water availability, and low and high temperatures, among others. The active function of these biomolecules is achieved thanks to post-translational modifications that involve structural changes and the proportion of specific amino acids, mainly acids. Under this premise, iterations have been placed on the enzymes produced by extremophile organisms, which, through evolutionary processes and the influence of biotic and abiotic factors, achieve molecular changes that permeate catalytic activity and, therefore, the permanence of the organism. Therefore, the catalytic activities of various enzymes isolated from commercial and wild organisms and active in biosolids and other substrates that are part of this complex mixture were determined (Table 2 and Table 3), such as those from *Trichoderma longibrachiatum* and *Aspergillus niger.*

The studies have shown the presence of degrading enzymes but also their activity against the components of biosolids. Pérez Soler [29] determines this process in two strains of *Aspergillus*. In the case of xylanases and cellulases, he observed a higher production of enzymatic activity in 7 days (150.4–174 U/kg of biosolid). The results were observed for up to 21 days in peroxidases, laccases, and esterases. In the case of *Penicillium martensii* NRC 345, laccase activity of 7.8 U/mg of protein has been determined, increasing to 39.52 U/mg when the organism is cultivated in the presence of wheat bran [30]. Esterase, laccase, and peroxidase assays in *Aspergillus sydowii* showed enzymatic activities of 1.5, 0.4, and 0.3 U/L in cultures supplemented with plant biomass [31]. In *Fusariu* C1BA.M3, laccases and lignin peroxidases have been evaluated for the degradation of textile dyes. The highest activity value for laccases is reported at 16 days with 308.64 U/L; in the case of lignin, peroxidase activity was reported up to 102.51 U/L [32]. Some oxidoreductases are not present in the degradation systems because the presence and ideal concentration of the expression inducer are necessary, as are cofactors such as iron, copper, and hydrogen peroxide, which intervene in the activation and function of the biological catalyst. Although the activity values are low compared to those reported for commercial enzymes, we must consider that these analyses have been carried out in a secretome where other proteins, compounds, and cellular residues that can interfere with the readings are present.

**Table 2:** Commercial enzymes produced by ascomycetes.

Enzyme	Organism	Identifier	Substratum	Reference
Xylanase	*Trichoderma viride*	EC 232-800-2	Hemicellulose	[33]
Xylanase	*Trichoderma longibrachiatum*	EC.3.2.1.8	Hemicellulose	[34]
Xylanase	*Aspergillus oryzae*	EC 253-439-7	Hemicellulose	[35]
Cellulase	*Aspergillus* sp.	EC 232-734-4	Cellulose	[36]
Cellulase Y-C	*Trichoderma viride*	N/A	Cellulose	[37]
Laccase	*Aspergillus* sp.	N/A	Phenolic and non-phenolic compounds	[38]
Laccase	*Aspergillus* sp.	EC.420-150-4	Phenolic and non-phenolic compounds	[39]
Lignin peroxidase	N/A	EC.1.11.1.14	Lignin	[40]
Manganese peroxidase	N/A	EC.1.11.1.13	Lignin, pigments, dichlorophen, bromoxynil, pentachlorophenol	[41]

N/A: not available.

As part of the degradation by biological treatment, competent enzymes (cellulases, xylanases, esterases, and peroxidases) have been isolated from ascomycete organisms for the degradation of various compounds found in active sludge. Over the years, some companies such as Sigma-Aldrich, Creative Enzymes, and MP BiomédicasTM have produced and marketed them for their applications (Table 2). In the same way, studies are carried out to evaluate the production of enzymes from newly isolated fungal organisms. Unlike commercial enzymes, this group is not yet in circulation since they are under molecular characterization; therefore, the sequences and information of each one can be acquired in the UniProt and NCBI databases (Table 3).

**Table 3:** Non-commercial enzymes produced by ascomycetes.

Enzyme	Organism	Identifer	Substratum	Reference
β-1,4- endoxylanase	*Aspergillus sydowii* *Aspergillus sydowii* CBS 593.65	A0A823A8R8	Hemicellulose	[42,43]
Glycoside Hidrolase	*Aspergillus sydowii* CBS 593.65	A0A1L9TUQ5	Hemicellulose	[44]
Endo xylanase	*Trichoderma* sp.	TXyn11A	Hemicellulose	[45]
Xylanase	*Trichoderma reesei* SAF3		Hemicellulose	[46]
Xylanase II	*Trichoderma reesei* RUT-C30	N/A	Hemicellulose	[47]
Laccase	*Aspergillus flavus*	N/A	Antibióticos, lignina, HPAs, plaguicidas, colorantes, lignina	[48]
Laccase	*Penicillium martensii* NRC 345	N/A	Antibiotics, lignin, HPAs, plaguicides, dyes	[30]
Laccase	*Podospora anserina*	N/A	Dyes and drugs	[49]
Laccase	*Fusarium* C1BA.M3	N/A	Textile dyes	[32]
Lignin peroxidase	*Fusarium* C1BA.M3	N/A	Textile dyes	[32]

N/A: not available.

#### 3.1.1. Fungal Enzymes Are Involved in the Treatment of Municipal Biosolids

The environmental services offered by natural resources may involve obtaining carbon and energy sources, the availability and recharge of hydrogeological resources, and recycling organic matter.

Notably, in materials biodegradation processes, mesophilic and extreme environments provide an endless number of macro- and microorganisms with particular and exciting characteristics to produce various enzymes that intervene in biotechnological processes as degradation routes. Through group and individual analysis, these biologically active macromolecules have shown efficiency in the stabilization, degradation, and mineralization of recalcitrant organic and inorganic compounds. The application of these catalysts includes the formation of products such as food additives, paints, textiles, and preservatives, as well as the degradation of plant matter, pesticides, dyes, and complex mixtures such as biosolids resulting from wastewater treatment.

In this field, enzymes have exhibited versatility since they can present intracellular and extracellular activity on various bonds and compounds. Based on the above, we can find phosphatases, hydrolases, polymerases, lyases, oxidoreductases, and transferases, among others [50].

Organisms include the ascomycetes of the genus *Cladosporium, Fusarium Trichoderma, Penicillium*, and *Aspergillus* [48,49,50,51,52,53], which present good expression of active enzyme cocktails in substrates such as cellulose, hemicellulose, lignin, polycyclic aromatic hydrocarbons, and pesticides. They are expected to be able to be active in complex systems such as biosolids. Activated sludge or biosolids are considered the final residue of a wastewater treatment process. Its content, vegetable and animal organic matter, microorganisms, and aromatic residues have been reported. For its treatment, handling, and final disposal, physical (incineration), chemical (alkaline stabilization), and biological (enzymatic digestion) systems have been implemented.

Some biological systems can synthesize hydrolase and oxidoreductase-type enzymes such as cellulases, xylanases, peroxidases, laccases, esterases, and mono- and di-oxygenases. The first two groups carry out the depolymerization by hydrolysis of polysaccharides such as cellulose and hemicellulose, and the rest can reduce aromatic compounds and their derivatives; in some cases, the fungal strains can synthesize lipases, proteases, and chitinases, by which they manage to eliminate pathogens by the degradation of membrane proteins and fatty acids, as well as chitin glucans arranged in the cell wall.

##### Cellulases

They are a group of macromolecules classified as glycosyl hydrolases (GH), CAZY, or carbohydrate-dependent enzymes, of which 173 families and 56 subfamilies are exhibited. Most of them present modular structural architecture made up of a peptide sequence without catalytic activity; they also present the carbohydrate-binding module (CBM), which is made up of approximately 30 to 200 amino acids that are occasionally arranged in the central region and usually in the amino region and terminal carboxyl [54]. The MBC in these enzymes is associated with a flexible hinge composed of glycosylated amino acids such as serine (Se) and threonine (Tre). For the catalytic site, a nucleophilic amino acid is exhibited, which at optimum pH has a negative charge, and the second acts as a proton donor. Depending on the organism under study, these residues can be aspartic: glutamic, aspartic: aspartic, or two glutamates [54]. Cellulases have activity on substrates such as crystalline and amorphous cellulose. Hydrolytic activity breaks the bonds in the b-1,4 glycosidic position [55]. Endoglucanases and exoglucanases are present in this enzymatic group, cutting the chains into various sizes of oligosaccharides. The resulting products will then be the raw material for cellobiohydrolases, which hydrolyze them to yield different structurally simple sugars such as cellotriose and cellobiose, among others [56]. Finally, cellodextrins are dissociated into glucose monomers by the action of b-D-glucosidases. On average and in different plant substrates, species such as *Aspergillus fumigatus* AF293 reported a production of 78 CAZY enzymes, of which 46 belong to the glycosyl hydrolase family GH 1, 3, 5, 6, and 7 [57]. In the case of *Trichoderma reesei* Rut CL847 and C30, the analysis and quantification of the secretome revealed an expression of 40 and 70 proteins, respectively; approximately ten families were identified, some such as cellobiohydrolases, endoglucanases, and β-glucosidases [47].

##### Xylanases

They are characterized by being a group that has activity on different substrates and mainly on the heterogeneous chains of hemicellulose. Hydrolysis of xylan heteropolymers provides sugars such as D-xylose, D-galactose, D-mannose, and L-arabinose, among others [58]. Their activity as inducers of expression and a diverse set of xylanolytic enzymes that degrade arabinoxylans, glucomannans, xyloglucans, galactomannans, etc. Referenced xylanolytic enzymes with carbohydrase activity belong to the glycosyl hydrolases and are members of the carbohydrate-dependent group (CAZY). They are distributed in families A, B, F, G, and H for bacteria and fungi, respectively. Because they are macromolecules belonging to GH, an active site has been described, made up of an aspartic residue and glutamate, which help hydrolyze the β-1,4-glucosidic bond. Endo-β-(1,4)-d-xylanases primarily hydrolyze arabinoxylan by cleaving internal 1,4-β-d-xylosidic bonds randomly. Next, the β-xylosidases finish the hemicellulose depolymerization process and begin the degradation of the xylan polymers into D-xylose units [59]. The branched galactomannan polymers are hydrolyzed by endo-β-1,4-mannases, separating the mannose units, while the β-galactosidases are responsible for separating the D-galactose from the main chain. For its part, the xyloglucan heteropolymer is degraded by various enzymes such as α-L-arabinofuranosidases, α-xylosidase, α-fucosidase, and β-galactosidase, generating residues of D-arabinofuranose, D-xylose, L-fucose, and D-galactose [60]. The CE carbohydrate esterase enzyme family has various biological catalysts. Among them, those with activity to separate the accessory groups present in hemicellulose stand out. Feruloyl and acetyl are the chemical species that abound in the chains to facilitate depolymerization, as are enzymes such as feruloyl esterase (CE2) and acetyl xylan esterase (CE1). It breaks the ester bond that holds the acetyl group to the xylan chains (CE1). CE1 separates feruloyl by hydrolyzing the ester bond linked to xylose. In the same way, it achieves the hydrolysis of compounds such as p-nitrophenyl acetate, α-naphthyl acetate, triacylglycerol, and cuticular waxes (CE5).

##### Peroxidases

This enzymatic group has been classified based on post-translational modifications and the organisms synthesizing them. For this reason, peroxidases with and without heme groups have been described. Particularly, those in this group are also known as heme peroxidases and have been observed in different living systems [61]. Although the characterization of these catalysts shows important peculiarities due to their low specificity and oxidoreductase activity, they manage to catalyze different phenolic and non-phenolic compounds [62,63]. The oxidation and reduction of compounds are carried out using a bisubstrate system. The enzymes use hydrogen peroxide (H_2_O_2_) as an oxidizing agent and a second substrate as a reductant, which is oxidized by H_2_O_2_. Peroxidases can be synthesized by bacteria, fungi, plants, and animals. They are biotechnologically relevant because they can mineralize plant-based and synthetic compounds, for example, pesticides, drugs, polycyclic aromatic hydrocarbons, dyes, and lignin. In the particular case of the latter compound, ascomycete fungi of the *Aspergillus genus* synthesize and excrete three types of peroxidases: Manganese Peroxidase (MnP), Lignin Peroxidase (LiP), and Versatile Peroxidase (VP) [53]. Other species, such as *Trametes versicolor* BAFC2234, cultivated in the presence of phenol, nitrophenol, AzureB, and black liquor revealed, through secretome analysis, the presence of 3 MnP and a VP that degraded 2- and 4-nitrophenol and phenol by 43% [64]. In Mexico, specifically in the Yucatan Peninsula, Amézquita [65] reported the oxidoreductase activity of the *Phlebia floridensis* strain against different compounds such as aniline blue, methylene blue, brilliant blue, G250, R250, and malachite green. The strain managed to mineralize the compounds, and an enzyme identified as chlorine peroxidase is believed to be closely related to the degradation processes.

##### Laccases

Multicopper oxidases, also known as polyphenol oxidases, can be divided into three types. They are a group that is also expressed in most living beings. They have been studied for their application in transforming and degrading recalcitrant compounds. Depending on the required process, they can be used as signifiers and detoxifiers, bleaching agents for paper pulp, stabilizers for alcoholic beverages, detergents, textile bleaches, and bioremediation and elimination of xenobiotics, as well as bioelectric sensors [66]. Due to their metallic cofactor, they are classified as blue because they present copper, yellow does not present a copper-binding site, and white presents zinc, iron, and copper.

As part of their structural characteristics, blue laccases have a heme group located in the catalytic region. Specifically, they have four copper ions (Cu^2+^), one of class T1, two of class T2, and one of class T3. Its spatial organization allows the binding of oxygen, which acts as the final electron acceptor. The role of fungal laccases has not been fully described. However, their participation in synthesizing pigments and depolymerizing lignin by breaking the alkyl-aryl bonds has been verified. This activity allows the oxidation of aromatic alcohols typical of this molecule, as well as p-diphenols, polyphenols, polyamides, inorganic ions, and arylamines [67]. Their low specificity characterizes their enzymatic versatility, and they have also been reported as catalysts with polymerization, methylation, and demethylation capacities. These enzymatic groups have been found in strains such as *Aspergillus nidulans*, *Rhizoctonia solanis*, *Trametes versicolor, and Neurospora crassa*.

##### Esterases

They are classified as a subgroup within the carboxylic ester hydrolases. In the lite-rature, they can be found as ferulic acid esterases, cinnamic acid hydrolases, *p*-coumaroyl, and cinnamoyl esterases, which are favorably expressed by plants, animals, and microorganisms. In some cases, these enzymes intervene in the pesticide degradation pathway, breaking ester bonds, as in the case of organophosphates such as methyl parathion [68].

In the case of hemicellulose—specifically acetyl xylan and feruloyl esterase—cutinases, the former dissociates the acetyl and feruloyl groups from the xylan chains, respectively, and the latter degrades the waxes and fatty acids of the plant cuticle. A Ser residue has usually been observed in its active site, which generates a nucleophilic attack to break the bonds [69,70].

There are other enzymatic groups called intermediates, for example, cutinases. They are enzymes with esterase-lipase activity with a catalytic triad formed by a nucleophile that can be serine, cysteine, or aspartic acid. Due to their folding, acid and histidine are found in the a/b family, where lipases, esterases, proteases, dehalogenases, and epoxide hydrolases are present [71]. For the specific case of cutinases and lipases, these managed to degrade long chains of fatty acids of animal, vegetable, bacterial, and fungal origin, for which they are used in various industrial processes such as obtaining biodiesel, modification of oils and fats, synthesis of aromatic substances, degradation of pesticides, phthalates, and synthetic polymers [72,73].

The organisms that managed to provide science with a cocktail rich in this enzymatic group are biotechnologically crucial because they are an alternative for various processes, including biodegradation. Fusarium oxysporum and Fusarium sp. are strains studied in the degradation of phthalates such as dihexyl phthalate (DHP) and di-(2-etihexyl)-phthalate (DEHP), which are used in the generation of various products for human use such as paints, pastes, paper, shoes, toys, etc. [74]. Due to their vast industrial distribution, they have been found in edaphic and water bodies, which causes contamination and intoxication due to water intake. Synthetic polymers, especially biodegradable ones, are not exempt from these organisms; fungal species such as Aspergillus oryzae and Fusarium solani have been outlined to describe the routes that are carried out for the biodegradation of these materials through the expression of cutinases and esterases specialized in hydrolyzing various synthetic esters of this nature. Likewise, genera such as Alternaria, Trichoderma, and Venturia, among others, have been studied based on the previously selected processes.

##### Challenges of Using Fungal Enzymes

The use of enzymes means hard and consecutive work to be able to present a functional, innovative product or one with improvements in its characteristics. Its isolation can generate a challenge, since we can find two groups: those with reduced production but high activity, and those with high production volume but low activity [75].

The abundance of the synthesis is also influenced by the presence of expression inducers, and knowing the concentration and nature of these molecules is of vital importance since a variety of enzymes can be blocked or obtained. For catalytic functions, peroxidases are one of the most complex groups to obtain since they catalyze reactions using hydrogen peroxide, a toxic compound for cells that is not very abundant.

The massive production of any enzymatic group and its molecular characterization can take months or even years of work; the difficulty lies in generating suitable specific cultures with wild or mutant strains. Finally, the search, isolation, identification, and commercialization of biotechnologically interesting enzymes, along with time constraints, competition, updating, and budget, are the biggest challenges for many researchers in developing countries.

### 3.2. Fungal Enzymes for Degradation of Polycyclic Aromatic Hydrocarbons

Polycyclic aromatic hydrocarbons (PAHs) comprise two or more fused rings with different structural configurations produced by incomplete combustion from natural and anthropogenic activities [76,77]. These xenobiotics represent a fraction of petroleum-derived hydrocarbons and constitute modern society’s energy source. PAHs are hydrophobic, possess very high boiling and melting points, and have a low vapor pressure. Their persistence in the environment is mainly due to their low solubility in water [78].

One of the most widely employed technologies for PAH remediation is biological processing. Specifically, the degradation of these pollutants by fungi has been thoroughly analyzed in state-of-the-art literature [79]. Most of these microorganisms cannot use these compounds as their sole source of carbon and energy; however, they can metabolize them to less toxic products and sometimes to CO_2_. Fungi mainly perform PAH degradation through monooxygenase enzymes [80].

Particularly in ligninolytic fungi, the enzymes LiP, MnP, and laccases oxidize PAHs and transform them into diphenol intermediates that are subsequently oxidized to quinones. These ligninolytic enzymes generate water-soluble polar compounds after the ligninolytic compounds’ catalytic cleavage and aromatic compounds’ catalytic cleavage, which become available for fungal metabolism [81]. Punnapayak et al. [82] demonstrated the degradation of 16 PAHs by the laccase enzyme produced by the white-poor fungus *Ganoderma lucidum* Chaaim-001 BCU. This enzyme completely degraded anthracene and benzo[a]pyrene, using 1-hydroxybenzotriazole as a redox mediator. Acevedo et al. [83] evaluated PAH degradation by *Anthracophyllum discolor*, a fungus isolated from the forest of southern Chile. The authors attributed the degradation of these organic compounds in Kirk medium to synergistic effects between PAHs or possible comet metabolism. The highest PAH degradation values were phenanthrene (62%), pyrene (60%), benzo[a]pyrene (75%), and anthracene (73%). Wirasnita and Hadibarata [84] analyzed biomass and enzyme production during the fluoranthene biodegradation process by *Pleurotus pulmonarius* F043. The extracellular ligninolytic enzyme system of the fungus was directly associated with the biodegradation of the xenobiotic, with laccase being the enzyme responsible for this decomposition process. The metabolites identified in fluoranthene degradation were naphthalene-1,8-dicarboxylic acid and phthalic acid.

Non-ligninolytic fungi produce enzymes similar to cytochrome P450 monooxygenase, which oxidizes PAHs and induces the formation of arene oxide and water; in addition, arene oxides, through non-enzymatic rearrangement, lead to the formation of phenols, which are conjugated with xylose, glucose, and gluconic acid [85]. These fungi can incorporate PAHs and their derivatives into their plasma membranes and oxidize and metabolize them into non-toxic products. However, accumulation of PAHs in the membrane can be toxic to the fungal cell [86]. Reyes-César et al. [87] used crude oil and PAH as a carbon source to grow and develop nine strains of non-ligninolytic fungi to demonstrate their potential for removing PAH from contaminated soils. The strains with the highest phenanthrene and pyrene degradation were *Aspergillus terreus*, *Talaromyces spectabilis*, and *Fusarium* sp., with 21% after 12 days of processing.

Some fungal species can also produce biosurfactants to overcome the hindrance of less soluble PAHs, resulting in enhanced degradation [88]. However, limited studies have been reported on the mechanisms and pathways involved in PAH decomposition by mycoremediation [86,89].

### 3.3. Liquid State Bioconversion

One of the most attractive technologies for reducing the volume of biosolids is liquid-state bioconversion (LSB). The LSB process consists of multiple treatments, including bioseparation, biodegradation, and biodrying, which involve the conversion of organic compounds into high-value-added products (e.g., biomass for composting purposes) by the action of microorganisms [90].

Alam et al. [91] used sludge as the primary substrate to obtain ethanol by LSB with the yeast *Saccharomyces cerevisiae*. The results showed that after 48 h of processing, 9.8% ethanol was achieved. The highest percentages of chemical oxygen demand (COD) and heavy metal (chromium and copper) reduction (62%, 45%, and 68%, respectively) were achieved at 72 h of fermentation.

Sludge is composed of organic elements that can be transformed by fungal action. Fungi consume organic matter in acidic systems, limiting the development of bacteria [92]. In the bioconversion process, filamentous fungi reduce the volume of biosolids through the mycelium, which modifies their structure and increases their bioseparation and dehydration [93]. Several studies have shown that the excretion of enzymes by filamentous fungi also favors the decomposition of the substrate used in the sludge.

Binti [94] developed a bioconversion process using the filamentous fungus *Trichoderma reesei* Rut C-30 to produce cellulase from biosolids. A Plackett-Burman design was used to optimize the medium components. Statistical analysis showed that the combination of 1.5% cellulase, 0.5% peptone supplemented with 0.20% Tween 80, 0.25% KH_2_PO_4_, and 0.03% MgSO_4_ produced a maximum cellulase activity of 21.33 U/mL. The optimization results showed that the inoculum concentration conditions and LSB parameters significantly affected cellulase production.

Rahman et al. [95] employed the LSB technique in a large-scale bioreactor for sewage sludge treatment. The authors analyzed the bioremediation performance using a mixed culture of *Aspergillus niger* and *Penicillium corylophilum* fungi at different values of organic loading and hydraulic retention time (HRT) of three and ten days, respectively. The highest percentage reduction of COD, turbidity, and total suspended solids was obtained after ten days of HRT. The removal of volatile solids, a parameter related to water quality, was 0.72 at three days and 0.59 at ten days of HRT, respectively. This study demonstrated that treating sewage sludge with LSB using mixed fungi represents a promising and environmentally friendly technology for the biological treatment of wastewater on a commercial scale.

## 4. Lignocellulosic Materials as a Carbon Source in Biosolids Treatment

Biological processes for decontaminating natural and artificial systems require a carbon and energy source such as biomass, nitrogen cycle derivatives, carbon derivatives, and hydrocarbons, among others. Most of these compounds can be used in synthesizing organic and inorganic materials, which play a vital role in forming new biological macromolecules that the organisms of the system can use under study.

Plant matter, or biomass, is a mosaic made up of fatty acids, waxes, carbohydrates, and, in some cases, proteins. It is characterized by being present in a broad group of organisms, such as herbaceous plants, woody plants, and algae. The proportion and degree of polymerization are different depending on the plant species.

In the case of agricultural stubble products such as corn, sugar cane, rice husks, wheat straw, oat straw, agave fibers, sawdust, sorghum, etc., it is reported to be present in an average range of 1000 mg/kg. On average, cellulose percentages ranging from 34 to 44%, hemicellulose from 25 to 27%, and lignin from 18 to 20% are reported [74,96].

Since these materials are in higher proportion, they are expected to be the primary substrate in biological degradation. These macromolecules’ pentoses, ferulic acid, galacturonic acid, methyl groups, and aromatic alcohols serve as carbon sources, which are metabolized through different routes to obtain energy and carbon [97]. Therefore, plant biomass can serve as an essential resource for treating biosolids, where organisms take advantage of carbohydrates, increase biomass by cell division, and metabolically can be active for the degradation of other pollutants with greater structural complexity. The cells of agricultural plant residues present a superficial layer called the cuticle, which is provided with hydrophobic chains that give it structure and function. Its composition is cemented by 16- and 18-carbon grade acids; some examples are 10,16-dihydroxyhexadecanoic acid, 9,16- dihydroxyhexadecanoic acid, 9, 10- epoxy-18-hydroxyoctadecanoic acid, and 9,10,18- trihydroxyoctadecanoic acid [98].

We can also find cuticular waxes consisting of alkane groups, alcohols, esters, aldehydes, and long and short-chain esters [99]. The integral hydrolysis of the cuticle is partly supported by enzymes of the carbohydrate esterase (CE) family that aid in dissociating the chains by breaking the ester bonds that hold them together. Removal of the cuticle facilitates access to the internal chains. Moreover, once the organisms have colonized this substrate, the cellulose is then exploited, degrading it into small oligosaccharides to finally obtain reducing sugars that will later be transported to the intracellular region [100]. In the case of hemicellulose, degradation is more complex because different enzymes (cocktails) are necessary. Due to its heterogeneous and branched nature, it needs an arsenal for its decomposition. The exciting thing about the macromolecule is the presence of various sugars that can function as a source of carbon and energy and inducers of xylanolytic enzyme transcription [97,100,101]. As a final part of this lattice and in particular, lignin is the only aromatic component and the most recalcitrant of the three. Therefore, microorganisms will seek cellular strategies that do not demand more energy than is available to dissociate the radicals associated with the aromatic ring and hydrolyze phenol bonds successively.

When organisms do not present enzymatic systems for degradation, or these are inefficient, they can exploit other biomass resources; cuticle lipids will now be the primary target for those microorganisms and their metabolism, as well as some free carbohydrates and proteins. Mutualistic work between organisms has been observed in any environment, and biosolids as an artificial microenvironment are no exception. Based on the above, microbial consortiums promise an efficient and complete degradation of contaminants in complex mixtures, where lignocellulolytic organisms degrade plant matter, other aromatic groups, dyes, proteins, and lipids, among others. In the same vein, active metabolism permeates the presence of various functional groups for cellular anabolism, an essential part of manufacturing energy molecules and biological macromolecules involved in processes such as DNA replication, RNA translation, enzyme synthesis, and cofactors, cell division, and maintenance, among others. Once organisms ensure their environmental persistence, they take up carbon sources for cellular reserve systems. In particular, microorganisms such as bacteria, fungi, and lignocellulosic yeasts, or degraders of plant matter, are a relevant part of the necessary treatment of biosolids; due to their existence in the mixture, we can inactivate various highly toxic polluting compounds for the disposal and final revaluation of materials with low or no danger.

## 5. Recent Trends

### 5.1. Recent Trends in Enzymatic Treatment

Biological systems—the expression and repression of enzymes of biotechnological interest—have emerged as recurring models to resolve environmental damage caused by the addition of pollutants and abundant biological materials.

In the case of extreme enzymes, catalysts are sought that can work in rigorous systems with parameters of pH, temperature, pressure, and salinity. Until today, organisms have been found that manage to synthesize them and also have evolutionary characteristics involved in post-translational modifications, structural changes, reduction, and abundance of charged, polar, and acid amino acids. Similarly, they show versatility in the degradation of polyurethane-based micro and nano plastics [102,103], as well as in the generation of new products such as synthetic polyester fabrics through enzymatic polymerization and the addition of poly (3, 4-ethylenedioxythiophene) [104]. Another relevant example is the esterases involved in the generation of chiral, anti-inflammatory drugs, and intermediate products. The synthesis of ferulic acid as an antioxidant, bleaching agents, biosensors for cholesterol detection, antitumor and anticancer agents, etc., [105].

Enzymes can be expressed naturally or heterologously, depending on where the coding sequence has been located, to later be inserted into prokaryotes and ascomycetes [106,107]. Based on the above, an isolated, efficient, and abundant production is sought to reduce costs and time in results, for which mutation processes have also been generated, where the change of amino acids in the active site sometimes achieves a greater catalytic reaction speed, thermal stability, and tolerance to salinity, which allows them to continue active in catabolism or anabolism depending on the process to be carried out [108,109,110].

### 5.2. Recent Trends in Biorefinery Approach

As mentioned before, in order to modify the substrates and make them suitable for easy utilization by microbes in the anaerobic digestion process, biosolid pretreatment is needed. Higher solubility and surface area of the pretreated substrates enable enzymatic action during anaerobic digestion, favoring a higher biodegradability rate.

Digested biosolids can usually be discarded into landfills or spread as fertilizer. When treated appropriately, returning biosolids to farmland completes a natural nutrient cycle. In this way, biosolids nutrients such as nitrogen and phosphorus are returned to the soil. However, recent trends have shown that rather than discarding the biosolids, the waste hierarchy is needed. According to Uthirakrishnan et al. [9], the three R’s of waste consist of Reduce, Reuse, and Recycle which is a useful approach to make biosolids disposal safer by the anaerobic digestion process. Based on the procedures—reduction of biosolid volume, development of reusable byproducts—biogas (a renewable energy source), electricity, and reuse of leftover compost (used as fertilizer)—according to our opinion, the three R’s approach is suitable to apply to improving waste biomass disposal through biological developments that focus on the transformation of waste resources into value-added products.

In this renewable context, Duan et al. [111] suggested the biorefinery approach for biowaste conversion to value-added by-products, considering lignocellulosic materials as a carbon source. In fact, biomass has become an important resource for energy generation worldwide. However, two issues need to be addressed. First, biomass should be valued enough to increase its potential uses. Second, updated policy regulations pertaining to the sustainable use of biomass are needed. Policy that favors the sustainable use of biomass to produce energy and bioproducts under the biowaste biorefinery [112] approach to contribute to a low-carbon, cleaner environment, privileging on the one hand the recovery of waste and, alternatively, the obtaining of value-added by-products. For example, among the various biorefinery-based products, the fastest-growing commodity is bioplastics, which will increase by about 16.6% by 2025. Currently, bioplastics market growth is at 10% annually, covering approximately 10–15% of the total plastics market [113]. The biomass vaporization as well as policy act as a driver to produce bioenergy, another value-added product, and is a combination seen only in a few countries, such as Germany and Spain. In low-economy countries, research is needed as a driver to promote the efficient use of biomass. In this sense, participatory research plays an important role in countries with large amounts of biomass that are undervalued. In particular, participatory research handed over from the researcher to local people (usually from rural areas) can produce insights for policy and may challenge the assumptions on which bioenergy policy frameworks and the bioeconomy are based.

## 6. Conclusions

Important attention has been paid to the search for and improvement of processes for the treatment of biosolids. Unfortunately, in some cases, its application can generate large implementation, manufacturing, and maintenance costs; however, secondary compounds may be present in the treatment train that compromise both human and environmental life and health. Due to the above, biological systems, especially enzymes characterized by mesophilic and extreme fungal organisms, mark a fundamental gap towards their implementation as an alternative methodology for the decontamination and biodegradation of complex mixtures such as activated sludge.

Although the scientific community is involved in finding efficient, versatile, and robust biological macromolecules that can operate in rigorous systems, it has been difficult to generate enzymatic cocktails and methods that permeate production and produce short-term results. Due to the above, molecular biology is committed to some recombinant expression technologies, for which we could have an alternative in the generation of green technology.

One of the most attractive technologies for reducing the volume of biosolids is liquid-state bioconversion, which involves the conversion of organic compounds into high-value-added products by the action of microorganisms. These value-added products are the result of a biorefinery approach for biowaste conversion, in which various biorefinery-based products are rapidly growing as commodities (e.g., bioplastics), but also energy from biomass.

## Data Availability

Data supporting reported results can be found from authors as request.
